# Nano zinc oxide (ZnO) foliar nutrition improves yield, antioxidant defence, and zinc content in rice grown under Zn-deficient soil

**DOI:** 10.1186/s11671-026-04662-y

**Published:** 2026-05-27

**Authors:** Gobinath Rajendran, Humera Quadriya, Vijayakumar Shanmugam, Manasa Vakada, Surekha Kuchi, Sanjeeva Rao Durbha, Latha PC, Brajendra Parmar, Bandeppa Sonth, Prasad Babu MBB, Kumaresan Palaniappan, Sundaram Raman Meenakshi

**Affiliations:** 1 Division of Crop Production, ICAR-Indian Institute of Rice Research, Rajendranagar, Hyderabad, 500030 India; 2 Directorate of Open and Distance Learning, Tamil Nadu Agricultural University, Coimbatore, 641003 India

**Keywords:** ZnO nanoparticles, Biofortification, Zinc uptake, Zn harvest index, ZnSO_4_

## Abstract

Conventional Zn fertilisers have poor use efficiency (< 5%), whereas the high reactivity and bioavailability of nano zinc oxide (ZnO NPs) offer a potential and efficient Zn carrier. With this background, the present study compared the efficacy of ZnO NPs with bulk ZnO and zinc sulphate (ZnSO_4_) on improving seed germination, crop performance, and Zn fortification under both in vitro and field conditions. In an in vitro experiment, rice seeds were treated with bulk ZnO (500, 1000, and 1500 mg L^− 1^) and ZnO NPs (50, 100, and 150 mg L^− 1^) under variable soaking durations (24 and 48 h), with an untreated control. ZnO NPs @ 150 mg L^− 1^ treatment significantly enhanced germination percentage and seedling vigour compared to bulk ZnO. Based on these results, a field experiment was conducted to validate the performance of ZnO NPs under field conditions. Treatments included a control, bulk ZnO (500, 1000, and 1500 mg L^− 1^), ZnO NPs (150, 250, 350, and 500 mg L^− 1^), and ZnSO_4_ (0.5%), applied as foliar sprays at flowering and grain-filling stages. ZnO NPs at 150 and 250 ppm increased grain yield by 15% and 21%, respectively, while ZnSO_4_ increased yield by 27% over the control. Nano ZnO also enhanced chlorophyll *a* and *b* contents by 26–57% and 37–50%, respectively. Root length increased substantially at the optimal ZnO NPs (150 ppm) concentration but declined slightly at higher concentrations. Grain Zn content improved by increased by 1.53 fold with 150 ppm ZnO NPs, accompanied by higher physiological efficiency and Zn harvest index. Though ZnSO_4_ resulted a relatively higher yield increase, foliar application of ZnO NPs demonstrated improved zinc accumulation and use efficiency at lower application rates, indicating their potential as an efficient alternative strategy for zinc nutrition in rice under Zn-deficient soils.

## Introduction

Intensive agricultural practices and the imbalanced use of macronutrients (N: nitrogen N, P: phosphorus, and K: potassium) without corresponding micronutrient supplementation have led to a critical increase in micronutrient deficiencies in soils, specifically zinc (Zn), iron (Fe), manganese (Mn), and copper (Cu) [1]. Among these, Zn deficiency is the most widespread, driven by soil factors such as high pH, salinity, CaCO_3_ content and low organic matter content. Globally, Zn is deficient in about 50% of cereal-growing soils [2], which translates directly to lower Zn content in edible crops and contributes to severe human malnutrition. Alarmingly, 17% of the world population (1.1 billion people) are at risk of Zn deficiency related health issues, including premature deaths, immune dysfunctions, neuro-behavioural disorders, etc [3]. The problem is pressing in regions like India, where approximately 38% of soils are Zn-deficient, making it a major yield-limiting nutrient in staple systems like rice cultivation [4].

A major challenge to improving Zn content in crops is the poor synchronisation between the release of nutrients from conventional fertilizers and the actual high nutrient demand periods during the crop’s critical growth stages. This temporal mismatch results in remarkably low nutrient use efficiency, often ranging between 2 and 5%, as only a small fraction of applied nutrients is available for plant uptake [5]. Specifically for Zn, soil application using conventional sources like zinc sulfate (ZnSO_4_) frequently leads to rapid fixation and formation of insoluble, plant-unavailable compounds in the soil matrix [5]. These immobilization causes widespread Zn-deficiency in crops and grains, sometimes leading to yield losses of up to 30% in cereal crops even without visible symptoms [6]. This challenge is compounded by indiscriminate use of P fertilizers, improper application methods, cultivation of nutrient-exhaustive crop varieties, and continuous cropping systems that accelerate the depletion of available Zn in soils [6]. To overcome these challenges, the adoption of suitable Zn sources and the efficient application methods are essential [7, 8]. While soil application has limited success in increasing grain Zn concentration due to soil fixation, foliar application has emerged as a viable alternative strategy to bypass soil constraints [9]. By allowing direct nutrient absorption through the leaf surface, foliar application significantly reduces nutrient losses and improves utilization. Several studies have confirmed that applying Zn at key growth stages, such as anthesis and grain filling, significantly enhances Zn accumulation in rice grains [10].

In recent years, nanotechnology has garnered significant attention for its potential to revolutionize agricultural input delivery systems. Nanomaterials, typically sized between 1 and 100 nanometers, exhibit unique physicochemical properties, such as high surface area, enhanced reactivity, and superior solubility [11, 12]. These nano material (nanofertilisers) offer an innovative approach to addressing this challenge by enhancing the efficiency of nutrient delivery and absorption in plants an dmking them promising candidate for agricultural applications, particularly in improving nutrient use efficiency [13]. The reduced particle size and large surface-to-volume ratio of these materials facilitate better interaction and absorption with plant tissues and soil particles. Among the various nanomaterials, zinc oxide nanoparticles (ZnO NPs) have emerged as a promising Zn source for crops. Studies have shown that ZnO NPs can be absorbed multiple fold efficiently than their bulk counterparts, highlighting their superior bioavailability [14]. When applied foliarly, nano ZnO enables efficient nutrient delivery, as the nanoparticles can easily penetrate the leaf surface, dissolve to release Zn^2+^ ions, and rapidly translocate to vegetative tissues [15]. Previous research across various crops, including rice [15], wheat [16], and *Brassica* [17] has reported that ZnO NPs can positively influence plant physiology, including increased chlorophyll content, enhanced antioxidant enzyme activities, improved yield, and Zn enrichment in grains [18, 19]. It is hypothesised that ZnO NPs, upon exposure, dissolve into Zn^2+^ ions, which are translocated to various plant organs, ultimately enhancing Zn accumulation and overall plant performance. Although ZnO nanoparticles offer several agronomic advantages, the environmental fate and potential risks associated with their application in agroecosystems remain inadequetly understood [20–22]. From both agricultural and ecological perspectives, a systematic evaluation of the efficacy of ZnO NPs on seed germination, seedling growth, crop performance, and Zn enrichment under field conditions is crucial. However, despite growing interest, comparative information on the performance of nano ZnO against conventional Zn fertilizers in Zn-deficient soils remains limited. Therefore, identifying appropriate Zn sources with higher bioavailability and assessing their Zn use efficiency under deficient conditions are essential steps in developing effective nutrient management strategies to combat Zn nutritional deficiencies in both plants and humans. Considering these aspects, a systematic study was conducted at the ICAR–Indian Institute of Rice Research, Hyderabad, India, during 2022 to evaluate the efficacy of ZnO NPs, bulk ZnO, and conventional ZnSO_4_ in enhancing seed germination, seedling growth, physiological responses, yield attributes, and Zn fortification of rice grown under Zn-deficient conditions.

## Materials and methods

This study consisted of three phases, namely (2.1) *synthesis and characterisation of* ZnO NPs, (2.2) *laboratory germination test using synthesised* ZnO NPs, and (2.3) *field-level evaluation* of ZnO NPs via foliar application on rice crop.

### Synthesis and characterisation

ZnO NPs were chemically synthesised using the acetate method, following the established protocol described by Rajendran et al. [23]. All chemicals and reagents utilized for both their synthesis and subsequent soil and plant analyses were of analytical grade and procured from Sigma Aldrich, India. The synthesised ZnO NPs were then characterised using their suspension to confirm their key physicochemical properties. X-ray diffraction (XRD) was employed to determine the crystallinity, shape, and size. Scanning electron microscopy (SEM) and transmission electron microscopy (TEM) were used to ascertain the morphology and size. Electron Dispersive X-ray (EDX) spectroscopy was used to verify the elemental composition and purity.

### Seed germination study

**Fig. 1:**
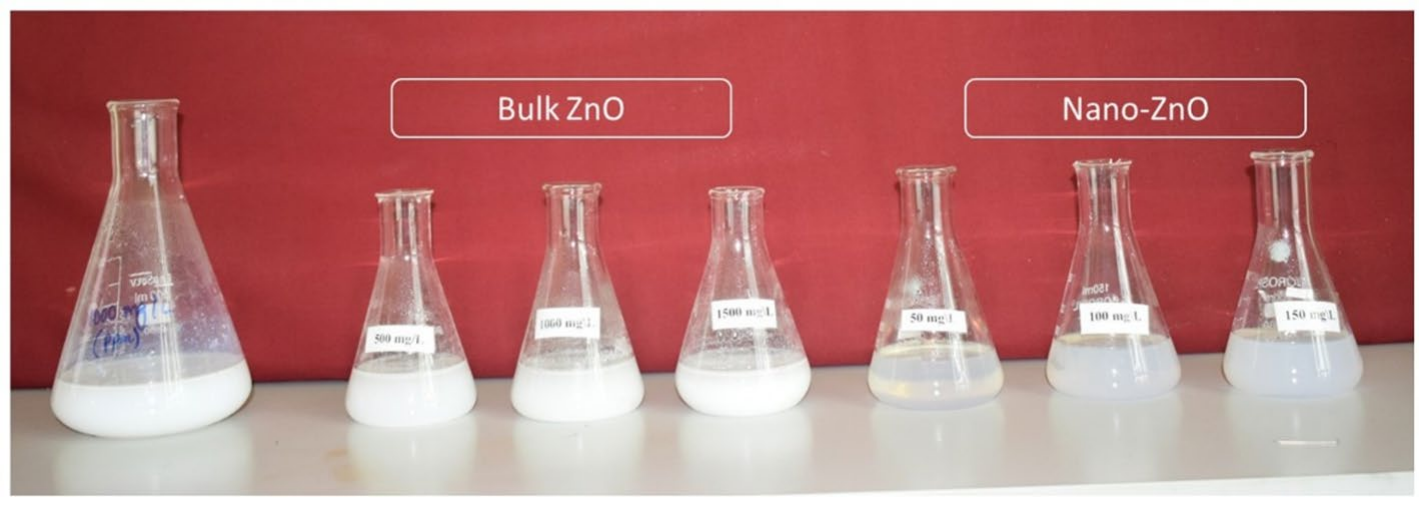
Suspension of ZnO nanoparticles in different concentrations

To evaluate the effect of ZnO NPs concentration, rice seeds (*var*. DRR Dhan-42) were subjected to an in vitro study. Ten grams of seeds were surface-sterilised with 2% sodium hypochlorite for 10 min and rinsed twice with distilled water. A stable suspension of ZnO nanoparticles was prepared using 0.2% sodium dodecyl sulfate (SDS) as a dispersing agent. The same concentration of SDS was applied uniformly across all treatments, to improve nanoparticle dispersion and prevent aggregation in the aqueous medium (Fig. 1), and seeds were soaked in this suspension for 24 and 48 h under different ZnO treatments, viz., control, bulk ZnO (500, 1000, 1500 mg L^− 1^), and nano ZnO (50, 100, 150 mg L^− 1^). Seedling length was recorded in centimetres (cm) using a wooden scale, and the germination percentage and seedling vigour were then assessed on the 5th day and 7th day after soaking, using the following formulas

$$\:\mathrm{G}\mathrm{e}\mathrm{r}\mathrm{m}\mathrm{i}\mathrm{n}\mathrm{a}\mathrm{t}\mathrm{i}\mathrm{o}\mathrm{n}\:\mathrm{p}\mathrm{e}\mathrm{r}\mathrm{c}\mathrm{e}\mathrm{n}\mathrm{t}\mathrm{a}\mathrm{g}\mathrm{e}\:\left(\mathrm{\%}\right)\:=\frac{No.of\:seeds\:germinated}{Total\:seeds\:kept\:for\:gemination}\times\:100$$

$$\:\mathrm{S}\mathrm{e}\mathrm{e}\mathrm{d}\:\mathrm{V}\mathrm{i}\mathrm{g}\mathrm{o}\mathrm{r}\:\mathrm{I}\mathrm{n}\mathrm{d}\mathrm{e}\mathrm{x}=\:\mathrm{\%}\:\mathrm{G}\mathrm{e}\mathrm{r}\mathrm{m}\mathrm{i}\mathrm{n}\mathrm{a}\mathrm{t}\mathrm{i}\mathrm{o}\mathrm{n}\times\:\mathrm{M}\mathrm{e}\mathrm{a}\mathrm{n}\:\mathrm{L}\mathrm{e}\mathrm{n}\mathrm{g}\mathrm{t}\mathrm{h}\:(\mathrm{S}\mathrm{h}\mathrm{o}\mathrm{o}\mathrm{t}+\mathrm{R}\mathrm{o}\mathrm{o}\mathrm{t})$$

### Field experiment

**Fig. 2:**
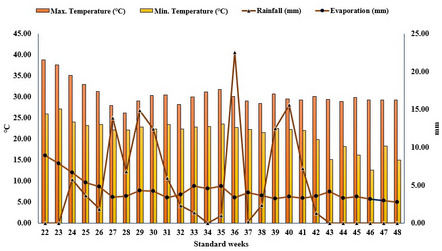
Weather conditions during the crop growth period (June to November 2022)

A field experiment was conducted during the 2022 wet season (*kharif*) at the experimental farm of ICAR–Indian Institute of Rice Research, Hyderabad (17.3201° N, 78.3939° E) to evaluate the effects of varying concentrations of ZnO NPs, bulk ZnO, and ZnSO_4_ on rice performance. Safety barriers were installed around the ZnO nanoparticle-treated plots to minimize the potential dispersion/drift of nanoparticles through the air and to ensure containment within the experimental area. The experimental soil had a pH of 8.1, an EC of 0.31 dS m^− 1^, 0.19% of organic carbon, and a deficient level of DTPA-extractable Zn (0.48 mg kg^− 1^), confirming its Zn-deficient status. The soil was classified as *Typic Ustifluvent*. Seedlings of rice variety DRR Dhan-42 (18–20 days old) were transplanted at a spacing of 20 × 15 cm. A basal fertiliser dose of 100:60:40 kg N: P: K ha^− 1^ was applied with N being split into three applications (50, 25, and 25 kg ha^− 1^ at basal, tillering, and panicle initiation, respectively). Weather data, including temperature and rainfall, recorded throughout the crop duration is presented in Fig. 2. The experiment followed a randomized block design with three replications and nine treatments as follows, T_1_: ZnO NPs (150 mg L^− 1^), T_6_: ZnO NPs (250 mg L^− 1^), T_7_: ZnO NPs (350 mg L^− 1^), T_8_: ZnO NPs (500 mg L^− 1^), and T_9_: ZnSO_4_ (0.5%). Two foliar sprays were applied at flowering (45 days after transplanting-DAT) and grain-filling (65 DAT) stages. ZnO NP suspensions were prepared as described in Sect.  2.2, while bulk ZnO and ZnSO_4_ solutions were prepared in double-distilled water.

#### Measurement of chlorophyll content and plant defense enzyme activity

Plant samples were collected at 60 DAT (after the first spray at 45 DAT) to analyze chlorophyll content and key defense enzymes, viz., superoxide dismutase (SOD), catalase (CAT), and peroxidase (POX). For chlorophyll analysis, 50 mg of fresh leaf tissue was extracted with 10 mL dimethyl sulfoxide (DMSO) followed by incubation at 35 °C for 4 h [24]. The concentrations of chlorophyll *a* and *b* were determined by measuring the supernatant absorbance at 665 and 663 nm, respectively. The resulting total chlorophyll content was quantified in mg g^− 1^ fresh weight.

$$\:Chl\:a=12.7x\:D663-2.69\:x\:D645\:x\frac{V}{\left(1000\:x\:W\right)}$$

$$\:Chl\:b=22.9\:x\:D645-4.68\:x\:D663\:x\frac{V}{\left(1000\:x\:W\right)}$$

$$\:Total\:Chlorophyll\:\left(a+b\right)=20.2\:\left(D645\right)+8.02\:\left(D663\right)\:x\:\frac{V}{\left(1000\:x\:W\right)}$$

For plant enzyme estimation, 0.1 g of leaf tissue was ground in liquid nitrogen to inhibit proteolytic activity. The resulting grounded material was then homogenised in 10 mL of 0.1 M phosphate buffer (pH 7.5). The homogenate was centrifuged at 15,000 rpm for 20 min, and the resulting supernatant was used as the enzyme extract [25].

#### Superoxide dismutase (SOD) activity

SOD activity was determined by mixing 0.1 mL of enzyme extract with a reaction mixture containing 75 µM nitroblue tetrazolium (NBT) and 13.33 mM methionine in phosphate buffer (pH 7.8). Riboflavin was added to initiate the reaction, and the mixture along with controls, was exposed to fluorescent light (15 W). Absorbance was read at 560 nm, and one unit of SOD activity was defined as the amount of enzyme necessary to inhibit 50% of NBT reduction compared to the control [26].

#### Peroxidase (POD) activity

Peroxidase activity was measured according to the method described by Castillo et al. [27]. A 0.1 mL aliquot of enzyme extract was mixed with the reaction mixture, which consisted of phosphate buffer (pH 6.1), 16 mM guaiacol, and 2 mM H_2_O_2_. The increase in absorbance due to the formation of tetraguaiacol was recorded at 470 nm. Enzyme activity was expressed as µmol of tetraguaiacol formed per minute per gram of fresh weight.

#### Catalase (CAT) activity

Catalase activity was determined by mixing the enzyme extract with phosphate buffer (pH 7.0) and 12.5 mM H_2_O_2_. The reaction was initiated by adding H_2_O_2_, and the decrease in absorbance at 240 nm was recorded for a period of one minute.

#### Estimation of soil enzyme activities

Root-bound soil samples were collected to estimate the activities of dehydrogenase, acid phosphatase, and urease enzymes. Urease activity was determined using urea as a substrate [28]. Five grams of moist soil was treated with a 2% urea solution and incubated at 37 °C for 5 h. Following incubation, 50 mL of potassium chloride (KCl)-phenyl mercuric acetate (PMA) solution was added, the mixture was further shaken for 1 h, and filtered through Whatman No. 42 paper. The filtrate was then mixed with a colouring reagent and heated in a water bath, and the resulting coloured solution was read at 527 nm using a single-beam spectrophotometer (Systronics, India). Urease activity was expressed as mg urea hydrolyzed g^− 1^ soil h^− 1^ at 37 °C. Acid and alakialine phosphatase activity was determined using p-nitrophenyl phosphate (p-NPP) as the substrate [29]. One gram of moist soil was mixed with 4 mL of modified universal buffer (pH 6.5 and 11 for acid and alkaline, respectively) and 0.025 mM p-NPP, incubated at 37 °C for 1 h, and the reaction was stopped by adding 1 mL of 0.5 M CaCl_2_ and 4 mL of 0.5 M NaOH. Absorbance of the yellow color was measured at 440 nm and expressed as µg p-NPP hydrolyzed g^− 1^ soil h^− 1^. Dehydrogenase activity (DHA) was assessed using 0.3% triphenyl tetrazolium chloride (TTC) as the substrate. 5 g of moist soil was incubated at 30 °C for 24 h, followed by extraction with 40 mL acetone. The absorbance of the resulting formazan was measured at 546 nm, and DHA was expressed as µg TTC reduced g^− 1^ soil h^− 1^ [30].

#### Yield, zinc content and uptake in plants

The grain and straw yields were recorded at the physiological maturity stage at 14% moisture and subsequently stored for Zn estimation. For Zn analysis, a 0.5 g of the dried plant straw and grain samples was weighed into separate conical flasks and digested using 10 mL of diacid mixture (4:2 as HNO_3_ : HClO_4_) on a hot plate for two hours at 400 ℃. Following digestion, the resultant samples were diluted, and the final volume was made up to 50 mL using double-distilled water. The resultant solution was then analysed for Zn content using an Atomic Absorption Spectrophotometer (AAS) (Varian 240, Agilent, USA). The zinc uptake in rice plant parts was calculated as per the formula given below,

$$\:Zinc\:uptake\:\left(\frac{g}{ha}\right)=yield\left(\frac{kg}{ha}\right)x\:Zn\:content\:\left(\frac{mg}{kg}\right)/1000$$

#### Determination of root parameters

After harvesting the crop, root-related parameters were measured using WinRhizo software. The roots were carefully and thoroughly washed with distilled water to remove all adhering soil particles. The cleaned roots were then placed in a glass plate containing water and spread out using forceps before the software was run for measurement. Root length (cm) and root surface area (cm^2^) were documented in the analysis.

#### Estimation of Zn use efficiency

The different Zn use efficiencies were computed using the following formulae [31], $\:Physiological\:Efficiency\:\left(PE\right)=\frac{Total\:yield\:in\:Zn\:applied\:plot-Total\:yield\:in\:control\:plot}{Zn\:uptake\:in\:Zn\:applied\:\:plot-Zn\:uptake\:in\:control\:plot}$

$$\:Zn\:harvest\:index\:\left(ZnHI\right)=\frac{Zn\:uptake\:in\:grain}{Total\:Zn\:uptake\:\left(Grain+straw\right)}x\:100$$

#### Statistical analysis

Treatment means were analysed using the analysis of variance (ANOVA), and the least significant difference (LSD) was calculated at the 5% significance level (*p* ≤ 0.05) using SAS software (version 9.4, SAS Institute Inc., USA) to provide a comprehensive comparison of pairwise treatment means. Mean values were presented as mean ± standard error (SE). Significant differences among treatments were further identified using Duncan’s Multiple Range Test (DMRT) at *p* = 0.05, where different letters (e.g., ‘a’, ‘b’, ‘c’, etc.) denote statistically distinct mean groups in figures and tables for clearer interpretation. Pearson correlation analysis was employed to assess the relationships among all measured variables.

## Results

### Characterisation of the synthesised material

**Fig. 3:**
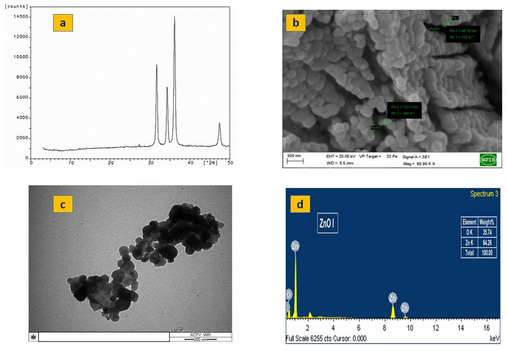
Images of ZnO nanoparticles **a**) X-ray diffraction (XRD) **b**) Scanning Electron Microscopy (SEM) **c**) Transmission Electron Microscopy (TEM) **d**) Electron Dispersive X-ray (EDX)

The XRD analysis confirmed the crystalline structure of the ZnO NPs, revealing an average crystallite size of approximately 43 nm estimated using Debye-Schrerrer equation (Fig. 3a). The SEM and TEM images revealed a flattened morphology for the NPs (Fig. 3b and 3c). Furthermore, SEM–energy dispersive X-ray spectroscopy (EDX) analysis confirmed the composition and purity of the ZnO NPs, indicating elemental compositions of 61% Zn and 32% Oxygen (Fig. 3d). Collectively, the characterization results demonstrated that the synthesised ZnO particles were within the nanoscale range and exhibited minimal agglomeration.

### Effect of ZnO nanoparticles on seed germination, seedling vigour, and seedling growth

**Fig. 4:**
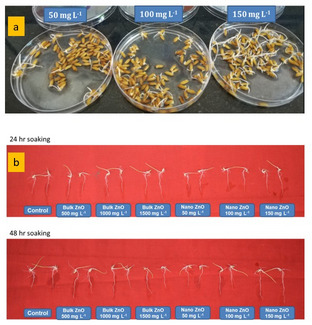
Effect of different sizes of ZnO on **a**) Seed germination and **b**) Seedling length of rice

Both bulk and nano ZnO significantly improved seed germination rates (Fig. 4; Table 1). Among all treatments, ZnO NPs at 150 mg L^− 1^ exhibited the highest germination rate, achieving 100% germination within 24 to 48 h of soaking, highlighting their strong positive influence on seed germination (Table 1). In contrast, bulk ZnO resulted in a lower germination rate of 90%, which was inferior to both the ZnO NPs treatment and the water-treated control, reflecting a reduction in germination percentage. Lower concentrations of ZnO NPs (50 and 100 mg L^− 1^) also exhibited high germination rates, with 97% and 95% recorded at 48 h, respectively, although these rates were slightly lower than the 150 ppm treatment. On the fifth day after soaking, seedlings treated with 150 mg L^− 1^ ZnO NPs exhibited the maxmium average seedling length of 9.56 cm, followed closely by 9.26 cm at 100 mg L^− 1^, both values surpassed the control. Specifically, at 150 mg L^− 1^ with a 24-hour soaking time, ZnO NPs increased seedling length by 15% after 5 days. Further growth improvements of 34% and 26.5% were observed on the 5th and 7th days, respectively, compared to the control. The results demonstrate the potential of ZnO NPs at an optimal dose (150 mg L^− 1^) to enhance early establishment and growth parameters more effectively than conventional Zn sources, without exhibiting phytotoxicity (based on both visual observation and growth measurements) during the initial five-day period, regardless of soaking duration.

**Table 1:** Effect of Zn source and dose on germination, seedling vigour index and seedling length of rice seeds

Treatments	Germination Percentage (%)		Seedling vigour index (24 h soaking)		Seedling vigour index (48 h soaking)		Seedling length (cm) (24 h soaking)		Seedling length (cm) (48 h soaking)	
	Soaking hours		5^th^ day	7^th^ day	5^th^ day	7^th^ day	5^th^ day	7^th^ Day	5^th^ day	7^th^ day
	24 h	48 h								
T_1_- Control	98^a^	92	925^a^	960	709^c^	860^b^	8.36^c^	9.76	7.83^b^	9.40^b^
T_2_- Bulk ZnO (500 mg L^-1^)	98^a^	100	823^c^	1009	764^b^	985^b^	9.40^a^	10.2	7.63^b^	9.86^b^
T_3_- Bulk ZnO (1000 mg L^-1^)	90^b^	100	810^c^	945	757^b^	948^b^	9.00^b^	10.5	7.56^b^	9.46^b^
T_4_- Bulk ZnO (1500 mg L^-1^)	90^b^	100	783^c^	950	777^b^	863^b^	8.70^b^	10.6	7.80^b^	8.63^b^
T_5_- ZnO NPs (50 mg L^-1^ )	98^a^	97	914^b^	1003	705^c^	961^b^	8.73^b^	10.2	7.26^b^	9.93^b^
T_6_- ZnO NPs (100 mg L^-1^)	100^a^	95	875^b^	1086	776^b^	910^b^	9.26^a^	10.9	8.16^b^	9.63^b^
T_7_- ZnO NPs (150 mg L^-1^)	100^a^	100	958^a^	1062	1050^a^	1187^a^	9.56^a^	10.6	10.5^a^	11.9^a^
LSD (0.05)	**3.32**	***NS***	**39.8**	***NS***	**97.3**	**161**	**0.30**	***NS***	**1.18**	**1.67**

LSD , Least significant difference; NS , Non Significant Values followed by different superscript letters within a column are significantly different at *P* ≤  0.05

### Effect of Zn sources on plant parameters - *Field study*

A field-level study was conducted to further evaluate the efficacy of different Zn sources (bulk ZnO, ZnO NPs and ZnSO_4_) on rice growth and yield parameters. The field-grown rice plants exhibited a significant response to Zn nutrition supplied *via* ZnO NPs and ZnSO_4_, particularly with plant height, grain and straw yield, and Zn concentration and uptake. Foliar application of nano ZnO and ZnSO_4_ markedly improved plant height (Table 2). Notably, ZnO NPs at 150 mg L^− 1^ resulted in the greatest enhancement, with an average plant height of 93.2 cm, outperforming both bulk ZnO and ZnSO_4_ treatments. However, increasing the nano ZnO concentration beyond 150 mg L^− 1^ did not lead to further gains, indicating a plateau effect at higher dosages. In comparison, ZnSO_4_ at 0.5% concentration produced a plant height of 88.2 cm, which was superior to higher concentrations of bulk ZnO and lower than ZnO NPs.

Differences in dry matter accumulation among the Zn treatments were not statistically significant (*p* > 0.05) (Table 2). However, grain yield responded positively to Zn application, although a significant difference was not observed. Compared to the control (no Zn supplementation), ZnO NPs at 250 and 150 mg L^− 1^, and ZnSO_4_ at 0.5%, increased grain yield by 17%, 13%, and 21%, respectively, when applied at flowering and grain-filling stages. Among all treatments, ZnSO_4_ resulted in the highest dry matter (straw+grain) accumulation (9.28 t/ha), contributing to a 15.7% increase in dry matter yield over the control. While ZnO NPs spray at 150 and 250 mg L^− 1^ registered 5.1% and 9.2% yield advantage over the control. Conversely, higher concentrations of nano ZnO (350 and 500 mg L^− 1^) led to marginal yield improvements of only 1.5% and 0.5% over control, suggesting potential toxicity or diminished efficacy at elevated doses. This declining trend suggests that above optimal rates of ZnO NPs, induces constraints like interfere with root metabolic activity, disrupt membrane integrity, and impair the uptake of other essential nutrients, ultimately reducing growth efficiency. Moreover, application of higher doses of bulk ZnO and ZnO NPs resulted in lower grain yields, comparable to those of the untreated control which highlight the importance of optimizing nanoparticle dosage, as excessive concentrations may not provide additional yield advantage. Straw yield showed minimal variation across treatments and did not differ significantly. Nonetheless, positive trends were observed with ZnO NPs at 250 mg L^− 1^ (1.1% increase) and ZnSO_4_ at 0.5% (6.7% increase) over control, indicating a modest benefit from these Zn sources in improving total biomass production. Though certain ZnO nanoparticle treatments showed numerical increases in yield compared with the control, some of these differences were not statistically significant (*p* > 0.05). Therefore, only treatments showing significant differences are interpreted as having a clear effect on yield. Previous studies have similarly reported that while low to moderate concentrations of ZnO nanoparticles enhance plant growth, higher concentrations may induce oxidative stress, inhibit root elongation, and reduce nutrient utilization efficiency. Therefore, the present results .

**Table 2:** Effect of Zn source and rate on yield parameters in rice crop *variety* DRR DHAN 42

Treatment	Plant height (cm)	Straw yield (t/ha)	Grain yield (t/ha)
T_1_-Control	84.4 ± 1.54^b^	4.40 ± 0.09	3.62 ± 0.14
T_2_-Bulk ZnO (500 mg L^− 1^)	84.4 ± 0.48^b^	4.28 ± 0.03	3.68 ± 0.14
T_3_-Bulk ZnO (1000 mg L^− 1^)	83.1 ± 1.05^b^	4.51 ± 0.32	3.70 ± 0.45
T_4_-Bulk ZnO (1500 mg L^− 1^)	83.2 ± 2.21^b^	4.31 ± 0.08	3.60 ± 0.30
T_5_-ZnO NPs (150 mg L^− 1^)	93.2 ± 0.87^a^	4.28 ± 0.04	4.17 ± 0.20
T_6_-ZnO NPs (250 mg L^− 1^)	86.9 ± 0.79^b^	4.45 ± 0.15	4.39 ± 0.16
T_7_-ZnO NPs (350 mg L^− 1^)	87.0 ± 2.04^b^	4.36 ± 0.10	3.79 ± 0.08
T_8_-ZnO NPs (500 mg L^− 1^)	83.1 ± 0.48^b^	4.30 ± 0.11	3.76 ± 0.10
T_9_-ZnSO_4_ (0.5%)	88.2 ± 1.07^ab^	4.69 ± 0.17	4.59 ± 0.35
**LSD (0.05)**	***5.0***	***NS***	***NS***

LSD , Least significant difference; NS , Non Significant. Values followed by different superscript letters within a column are significantly different at *P* ≤   0.05

Bold value indicates the level of significance corresponding to the respective parameters

### Antioxidant enzymes and chlorophyll content

Enhanced antioxidant enzyme activity was observed across treatments, with ZnSO_4_ expressing the highest response for all enzymes: SOD (33.4 EU min^− 1^ g^− 1^ fresh weight), CAT (90.9 µmol H_2_O_2_ reduced min^− 1^ g^− 1^ fresh weight), and POX (2.78 µmol tetra-guaiacol formed min^− 1^ g^− 1^ fresh weight) (Table 3). High-dose of ZnO NPs treatments (350 and 500 mg L^− 1^) also elevated enzyme activity, while lower concentrations (150 and 250 mg L^− 1^) resulted in modest SOD increases (23% and 14% over control, respectively). CAT activity remained statistically consistent across nano ZnO treated plants. Catalase activity was highest with ZnO NPs @ 500 mg L^− 1^ (87.3 H_2_O_2_ reduced/min/g wt), followed by 150 mg L^− 1^ (87.1 H_2_O_2_ reduced/min/g wt), 350 mg L^− 1^ (79.2 H_2_O_2_ reduced/min/g wt), and bulk ZnO @ 1500 mg L^− 1^ (79.1 H_2_O_2_ reduced/min/g wt), corresponding to increases of 17.5%, 17.2%, 6.6%, and 6.5% over the control (74.3 H_2_O_2_ reduced/min/g wt), respectively. The POX activity peaked with ZnO NPs @ 350 mg L^− 1^ (2.8 µmol tetra-guaiacol formed per minute per g), followed by ZnO NPs @ 500 mg L^− 1^ (2.7 µmol tetra-guaiacol formed per minute per g), showing 11% and 10% increase over control. Chlorophyll content was not significantly affected across Zn treatments. However, spray of ZnO NPs @ 500 mg L^− 1^ recorded the highest chlorophyll *a* (28.2 mg g^− 1^ FW^− 1^) and *b* (8.2 mg/g) levels. Relative to the control, ZnO NPs increased chlorophyll *a* by 26–57% and chlorophyll *b* by 37–50%. While conventional Zn source i.e., ZnSO_4,_ improved the chlorophyll *a* and *b* by 11% and 50%, respectively. These results suggest that increased activity of antioxidant enzymes may represent a protective response to maintain cellular redox balance under nanoparticle exposure, which could contribute to improved plant health and productivity.

**Table 3:** Effect of Zn source and rate on plant defence enzyme activity and chlorophyll content in rice leaves

Treatments	Superoxide Dismutase (EU/min/g fresh weight)	Catalase (H_2_O_2_ reduced/min/g fresh weight)	Peroxidase (µmol tetra guaicol formed/min/g fresh weight)	Chl a (mg g^− 1^ FW^− 1^)	Chl b (mg g^− 1^ FW^− 1^)
T_1_-Control	22.0	74.3^ab^	2.49	18.0	5.25
T_2_-Bulk ZnO (500 mg L^− 1^)	21.7	61.1^b^	2.44	20.1	6.00
T_3_-Bulk ZnO (1000 mg L^− 1^)	18.2	71.4^ab^	2.68	21.4	6.17
T_4_-Bulk ZnO (1500 mg L^− 1^)	24.6	79.1^ab^	2.56	18.6	5.93
T_5_-ZnO NPs (150 mg L^− 1^)	17.0	87.1^a^	2.64	22.8	7.69
T_6_-ZnO NPs (250 mg L^− 1^)	19.0	76.1^ab^	2.53	22.8	7.27
T_7_-ZnO NPs (350 mg L^− 1^)	25.9	79.2^ab^	2.76	22.6	7.17
T_8_-ZnO NPs (500 mg L^− 1^)	25.7	87.3^a^	2.71	28.2	8.18
T_9_-ZnSO_4_ (0.5%)	33.4	90.9^a^	2.78	19.9	7.88
**LSD (0.05)**	***NS***	**17.4**	***NS***	***NS***	***NS***

LSD, Least significant difference; NS, Non Significant. Values followed by different superscript letters within a column are significantly different at *P* ≤   0.05.

Bold value indicates the level of significance corresponding to the respective parameters

### Estimation of soil enzyme activities

**Fig. 5:**
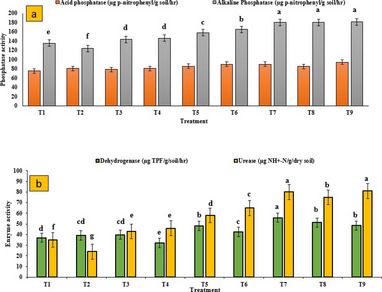
Effect of Zn application on soil enzyme activities - **a**) Acid and alkaline phosphatase and **b**) Dehydrogenase and urease activity. T1-control, T2-bulk ZnO (500 mg L^− 1^), T3- bulk ZnO (1000 mg L^− 1^), T4- ZnO NPs (1500 mg L^− 1^), T5 -ZnO NPs (150 mg L^− 1^), T6- ZnO NPs (250 mg L^− 1^), T7- ZnO NPs (350 mg L^− 1^), T8- ZnO NPs (500 mg L^− 1^) and T9- ZnSO_4_(0.5%)

Soil enzyme activities responded positively to Zn supplementation, with notable differences among the Zn sources (Fig. 5). DHA increased in all treatments except for bulk ZnO at 1500 mg L^− 1^ which recorded a 13.0% reduction compared to the control. The highest DHA (55.9 µg TPF g^− 1^ soil h^− 1^) was observed in soils treated with ZnO NPs at 150 mg L^− 1^, while the lowest activity (32.2 µg TPF g^− 1^ soil h^− 1^) was associated with the bulk ZnO at 1500 mg L^− 1^. Given the alkaline nature of the experimental soil, alkaline phosphatase activity (APA) was notably enhanced across Zn treatments, particularly with the addition of ZnSO_4_. Acid phosphatase and urease activities also increased, with the highest values observed at 94.0 µg p-nitrophenol g^− 1^ soil h^− 1^ and 81.0 µg p-nitrophenol g^− 1^ soil h^− 1^, respectively. In contrast, negative impacts on enzyme activities were observed with bulk ZnO, notably in the 1500 mg L^− 1^ treatment for dehydrogenase and the 500 ppm treatment for urease.

### Root parameters

The foliar application of Zn significantly influenced rice root morphological parameters. Both nano ZnO and ZnSO_4_ demonstrated a marked impact on total root length, surface area, and average root diameter compared to the untreated control (Table 4). The application of ZnO NPs at lower concentrations notably enhanced root development, with impressive increases of 141.5% in total root length, and 41.7% in both surface area, and average root diameter over the control. However, a linear decline in root length was observed with increasing nano ZnO concentrations of 250, 350, and 500 mg L^− 1^, corresponding to reductions of 48.2%, 28.5%, and 38.4%, respectively, from the peak value recorded at the lowest dose. Bulk ZnO treatments also promoted root growth relative to the control, with increases in root length ranging from 35.6% to 80.3% and surface area from 20.3% to 39.4%. Nonetheless, these improvements were less pronounced than those observed with nano-ZnO treatments.

**Table 4:** Effect of Zn application on different root parameters under field conditions

Treatments	Root length (cm)	Root surface area (cm^2^)	Average root diameter (mm)
T_1_-Control	98.1 ± 1.50^e^	356.0 ± 5.4^d^	0.12 ± 0.007^a^
T_2_-Bulk ZnO (500 mg L^− 1^)	152.1 ± 19.4^cd^	496.1 ± 23.7^ab^	0.20 ± 0.016^a^
T_3_-Bulk ZnO (1000 mg L^− 1^)	176.9 ± 7.20^bc^	432.3 ± 17.9^bc^	0.15 ± 0.004^a^
T_4_-Bulk ZnO (1500 mg L^− 1^)	133.0 ± 13.3^cde^	428.4 ± 5.8^c^	0.17 ± 0.004^a^
T_5_-ZnO NPs (150 mg L^− 1^)	236.9 ± 11.9^a^	504.3 ± 7.3^a^	0.17 ± 0.014^a^
T_6_-ZnO NPs (250 mg L^− 1^)	122.7 ± 9.8^de^	480.2 ± 7.3^abc^	0.16 ± 0.011^a^
T_7_-ZnO NPs (350 mg L^− 1^)	169.3 ± 20.1^bc^	455.7 ± 9.6^bc^	0.12 ± 0.006^a^
T_8_-ZnO NPs (500 mg L^− 1^)	145.9 ± 4.7^cd^	432.0 ± 20.5^abc^	0.14 ± 0.006^a^
T_9_-ZnSO_4_ (0.5%)	201.9 ± 6.5^b^	516.0 ± 30.3^a^	0.13 ± 0.004^a^
**LSD (0.05)**	**46.5**	**65.5**	**NS**

LSD, Least significant difference; NS, Non Significant. Values followed by different superscript letters within a column are significantly different at *P* ≤  0.05

Bold value indicates the level of significance corresponding to the respective parameters

### Zinc concentration and uptake in straw and grain

**Fig. 6:**
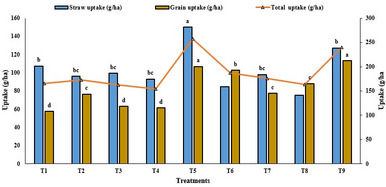
Zinc uptake in straw and grain samples of different treatments. T1-control, T2-bulk ZnO (500 mg L^− 1^), T3- bulk ZnO (1000 mg L^− 1^), T4-bulk ZnO (1500 mg L^− 1^), T5 -ZnO NPs (150 mg L^− 1^), T6- ZnO NPs (250 mg L^− 1^), T7- ZnO NPs (350 mg L^− 1^), T8- ZnO NPs (500 mg L^− 1^) and T9- ZnSO_4_ (0.5%)

Zinc supplementation significantly enhanced Zn accumulation in rice roots, straw, and grains (Fig. 6). Foliar application of nano ZnO at 150 mg L^− 1^ resulted in the highest grain Zn content, achieving a 53.4% increase over the control, followed by 0.5% ZnSO_4_ (47.3%), and ZnO NPs at 250, 350, and 500 mg L^− 1^ (44.0%, 41.0%, and 39.6%, respectively). Bulk ZnO treatments showed minimal improvement (ranging from 3 to 28%). Straw Zn concentration was also highest under the 150 mg L^− 1^ spray of ZnO NPs (35.1 mg kg^− 1^; representing a 35% over control). However, Zn accumulation declined beyond the 150 mg L^− 1^ threshold for nano ZnO, indicating an optimum level. In terms of Zn uptake at harvest, ZnO NPs at 150 mg L^− 1^ recorded the highest values (150 g/ha in straw, 107 g/ha in grain, and a total of 257 g/ha). This was followed by 0.5% ZnSO_4_ with 127 g/ha (straw), 113 g/ha (grain), and 240 g/ha (total). Nano ZnO and ZnSO_4_ treatments enhanced total Zn uptake by 55% and 45% over the control, respectively, with a 10% advantage observed for the nano Zn source over the sulfate form.

### Zinc use efficiency

**Fig. 7:**
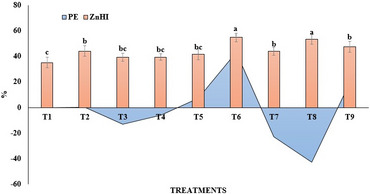
Influence of Zn sources on physiological efficiency (PE), and Zn harvest index (ZnHI) of field-grown rice crop. T1-control, T2-bulk ZnO (500 mg L^− 1^), T3- bulk ZnO (1000 mg L^− 1^), T4-bulk ZnO (1500 mg L^− 1^), T5 -ZnO NPs (150 mg L^− 1^), T6- ZnO NPs (250 mg L^− 1^), T7- ZnO NPs (350 mg L^− 1^), T8- ZnO NPs (500 mg L^− 1^) and T9- ZnSO_4_ (0.5%)

Differential application of Zn sources and rates caused pronounced variation in PE, while exhibiting negligible influence ZnHI (Fig. 7). ZnO NPs at 250 mg L^− 1^ achieved the highest PE (43.3 kg yield/g Zn) values, demonstrating enhanced efficiency in translating Zn to grain. However, optimal PE (7.2 kg yield/g Zn) was observed in lower ZnO NPs treatments. Notably, ZnO NPs treatments also recorded consistently higher ZnHI values across all doses, highlighting their superior translocation and partitioning efficiency in rice plants. Similarly, ZnHI values were largely invariant, implying that Zn translocation was stable across the treatments. Conversely, higher doses of ZnO NPs (350 and 500 mg L^− 1^) and all bulk ZnO treatments demonstrated a decline or negative trend in efficiencies, indicating potential negative effects at elevated Zn concentrations.

## Discussion

### Effect of ZnO NPs on seed germination and seedling length

Application of Zn *via* ZnO NPs significantly accelerated early seed germination, indicating a positive role in enhancing metabolic activity during the initial growth phases, which includes root and shoot development. The improved Zn bioavailability from ZnO NPs facilitated greater seed germination and seedling vigour, with the 150 mg L^− 1^ treatment showing optimal performance, particularly under 48-hour exposure conditions. In contrast, higher concentrations of ZnO NPs and bulk ZnO reduced germination percentages, likely due to agglomeration, which hinder nutrient uptake [15]. Furthermore, direct nanoparticle-seed interactions can lead to inhibitory effects depending on concentration and exposure time [32], suggesting that germination percentage is a reliable indicator of phytotoxicity response. While ZnO NPs enhanced seedling growth, maximum seedling length was recorded with 24-hour exposure, suggesting that prolonged exposure (48 h) may attenuate elongation due to potential nanoparticle stress. Similar inhibitory trends in seedling growth were observed with bulk ZnO, possibly due to its low solubility and limited Zn availability to support metabolic activities. This is consistent with previous reports on nano-ZnO effects across various crops, including tomato, wheat, and pea [33, 34].

### Efficay of ZnO on enhancing grain yield

The present study highlights the efficacy of ZnO NPs as a promising nano-fertilizer, capable of improving Zn nutrition, reducing oxidative stress, and promoting grain yield in rice, corroborating earlier findings [35]. Foliar application of Zn through nano form of ZnO and ZnSO_4_ markedly enhanced plant height, panicle development, and overall yield components, contributing to a substantial increase in grain yield. This improvement was closely linked to improved Zn availability during key critical stages, particularly flowering and grain filling, which are critical for assimilate partitioning and reproductive development. Two sprays of 150 mg L^− 1^ ZnO NPs yielded the highest grain output among all treatments, suggesting that this concentration optimises Zn availability without inducing toxicity. This aligns with findings from Adhikari et al. [36], who reported improved shoot and root biomass with optimal ZnO NP application. The positive yield response observed in this study supports earlier research by Dimkpa et al. [37]. in sorghum and Du et al. [38]. in wheat crops, and highlights the potential of ZnO nanoparticles as a scalable, efficient alternative to conventional Zn fertilisers for enhancing crop yield under field conditions.

### Effect of ZnO on plant physiological parameters

**Fig. 8:**
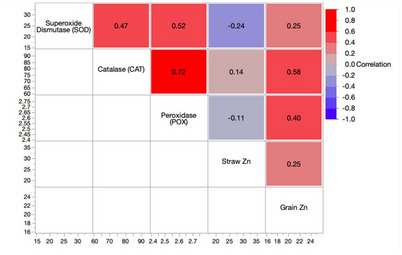
Pearson correlation analysis of enzyme activities and Zn content

The preliminary seed germination assay confirmed that nano form of ZnO exhibits no phytotoxicity and significantly promote early plant growth over bulk ZnO and untreated controls. Building upon these findings, field-level assessments demonstrated that foliar-applied Zn fertilizers, particularly ZnO NPs, significantly modulated plant defense responses, chlorophyll content, and soil enzymatic activity in rice though non significant differences observed in many of the parameters. But we observed, consistent response with the correlation analysis, which indicated positive associations with growth, nutrient uptake, and levels. At 60 DAT, ZnO NPs elicited higher activities of key antioxidant enzymes (SOD, CAT, POX), suggesting their role in enhancing the plant’s oxidative stress response. The enhanced activity of antioxidant enzymes observed at higher ZnO NPs concentrations may indicate the activation of plant defense mechanisms to nullify potential oxidative stress (maintaining cellular redox balance) induced by nanoparticle exposure. The 150 mg L^− 1^ of ZnO NPs treatment was notable for its lower SOD activity (17.0 EU/min/g) coupled with efficient hydrogen peroxide reduction, increased POX activity, and elevated chlorophyll *a* and *b* concentrations. Chlorophyll levels were markedly enhanced in ZnO NPs treated plants compared to both bulk ZnO and controls, indicating improved photosynthetic capacity. These findings align with prior studies by Mishra et al. [39] demonstrating Zn-induced upregulation of photosynthetic pigments and enzymatic defense systems. An increase in photosynthetic pigments might be attributable to amino acid complexes production induced due to Zn supply through nano formulaitons. comprising micronutrients and also substantial zinc ingestion enhanced the photosynthetic efficiency and restricted the gas exchange characteristics of the plant [40]. Positive correlations were observed between grain Zn content and enzymatic activities (r^2^= 0.25 for SOD, 0.58 for CAT, and 0.40 for POX), whereas negative correlations emerged between straw Zn and both SOD and POX (Fig. 8). This suggests a functional link between Zn uptake, antioxidant regulation, and productivity outcomes. Enhanced ROS-scavenging through CAT and POX activity, consistent with reports by Ahmed et al. [41] and Nazir et al. [42], reflects a coordinated antioxidative defense in response to ZnO NPs exposure in *Allium* sp. and wheat, respectively. This increase in ROS accumulation results from the upregulation of defence-related gene expression. Overall, nano ZnO application not only promoted photosynthetic pigment synthesis but also activated enzymatic defenses, contributing to improved stress resilience and growth in rice.

### Effect of ZnO nanoparticles on Zn concentration in plant parts

Foliar application of nano form of ZnO significantly enhanced Zn concentrations in rice straw, and grain compared to other Zn sources, attributed to their superior solubility and mobility. All Zn treatments increased Zn content in grain and root tissues over the control. Dual foliar sprays of 150 ppm nano ZnO raised Zn levels in straw and grain by 36.4% and 53.5%, respectively, indicating improved uptake and translocation to reproductive tissues. Foliar application at flowering and grain filling stages proved most effective, as nano-sized particles facilitate cellular penetration and targeted Zn accumulation with minimal losses. The increased penetration facilitates more efficient uptake of active compounds, resulting in improved effectiveness and minimizing the frequency of repeated applications [13]. These results align with Priester et al. [43] and Pandya et al. [44], who reported enhanced Zn uptake and root accumulation with nano-Zn treatments. ZnO NPs also outperformed ZnSO_4_·7H_2_O in crops like peanut, achieving comparable effects at doses ~ 15 times lower. Consistent with earlier findings [45], foliar application ensured sustained Zn supply during reproductive stages, leading to a 30.9–44.8% rise in grain yield and 39.6–53.5% increase in grain Zn content over control. Similar yield gains were reported by Sattar et al. [46] in wheat. Improved photosynthetic activity and nutrient efficiency under nano form of ZnO contributed to better grain filling and ZnHI. Overall, ZnO NPs demonstrate strong potential as nano-fertilizers to enhance grain yield and nutritional quality, aiding micronutrient enrichment and food security.

### Soil enzymes and root parameters

**Fig. 9:**
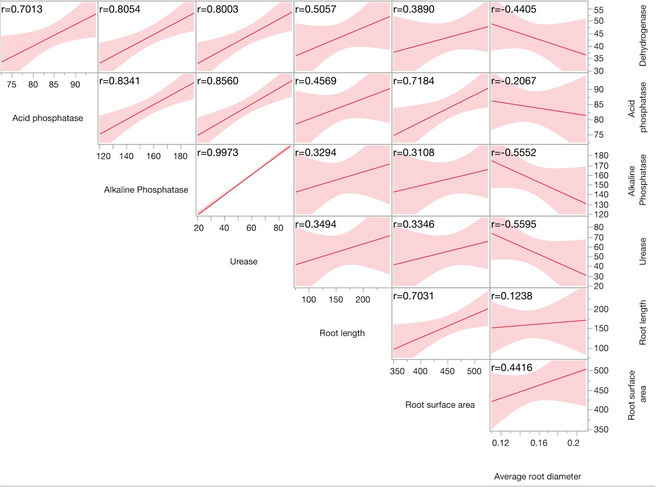
Correlation of root parameters with enzymes with line of fit, Pearson correlation coefficient, *P* < 0.05, ‘r’ values are correlation coefficients of data under study

Application of ZnO NPs led to a significant enhancement in soil enzymatic activities, including dehydrogenase (14.6–51.1%), acid phosphatase (14.1–20.0%), and urease (65.7–114%) relative to the control. These increases reflect improved microbial activity and nutrient turnover in the rhizosphere, likely driven by increased Zn availability and stimulation of root exudation. Such trends are consistent with earlier studies [47], although contrasting evidence [48] suggests potential inhibition at higher nano concentrations, emphasizing the importance of dosage optimization. Phosphatase enzymes, central to phosphorus mobilization under nutrient-limited conditions, were particularly responsive to moderate ZnO NPs doses. The overall stimulatory effect of Zn source i.e. ZnO NPs applied through folair mode appears to stem from their enhanced bioavailability and interaction with root-microbial systems, as the released Zn ions support microbial proliferation and enzyme secretion, promoting nutrient cycling. Cun et al. [49] reported enhanced dehydrogenase enzyme activity under foliar Zn application in sunflower, suggesting that Zn plays a synergistic role in stimulating soil microbial activity and associated biochemical processes. Scatter plot matrix analysis further established strong positive correlations between root traits and enzymatic activity. Notably, root length and surface area were significantly associated with dehydrogenase and acid phosphatase activities, while urease activity showed moderate correlations (Fig. 9). Roots are closely associated with plant nutrition and serve as the primary pathway for nutrient absorption. They also secretes acids to dissolute the fixed nutrients from the soils which potentially increased microbial proliferation in the root zone and nutrient cycling in soil, while excessive doses of bulk ZnO may inhibit key enzymatic functions. These results suggest that nano ZnO foster a more biologically active rhizosphere, enhancing both root development and microbial functioning, critical components for sustainable crop productivity.

### Zinc use efficiency

**Fig. 10:**
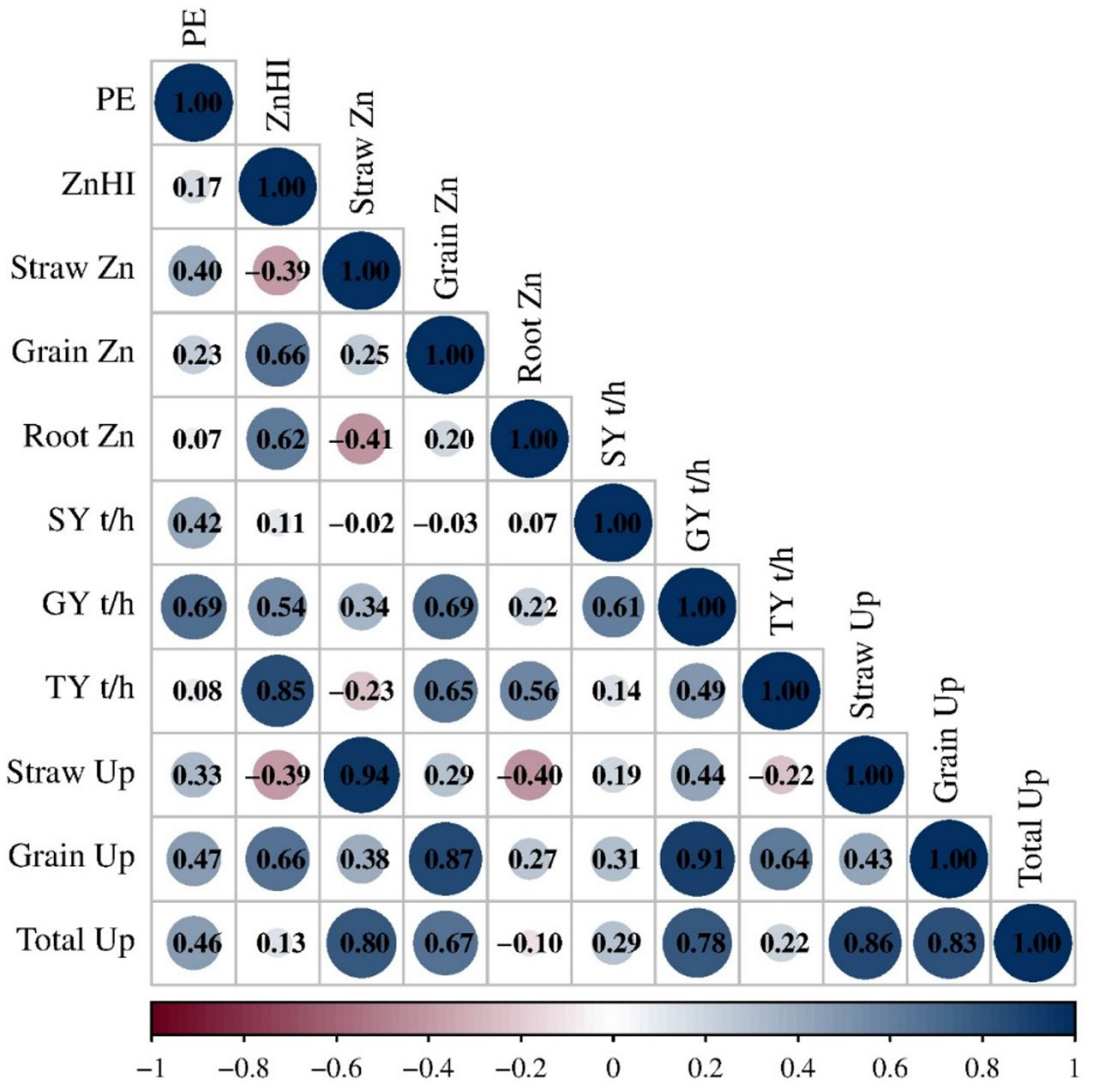
Pearson correlation analysis of use efficiencies with major traits

Zn use efficiency parameters, including biomass yield and nutrient uptake, improved markedly with Zn applied in nano form compared to bulk ZnO. Foliar application of ZnO NPs enhanced efficiency through controlled release, promoting microbial activity, enzymatic conversion, better root growth, greater nutrient mobility, and reduced losses. In contrast, higher doses of bulk ZnO (1000–1500 mg L^− 1^) showed negative PE values, indicating yield decline, poor solubility, Zn accumulation, and possible phytotoxicity. The decline in PE at elevated Zn concentrations reflected reduced chemical efficiency and potential nutrient loss. The pearson correlation heatmap of PE and ZnHI showed strong positive correlations with grain yield (r^2^ = 0.69, 0.54) and grain Zn content (r^2^ = 0.23, 0.66) (Fig. 10). Whereas ZnHI was positively correlated with all parameters except straw Zn. Grain Zn concentration showed a moderate positive association with root Zn concentration, suggesting an efficient translocation of Zn from the root to the grain. In contrast, straw Zn concentration displayed a negative correlation with yield traits, implying that higher biomass production may dilute Zn concentration in the vegetative tissues over the period. Contrarily, PE was moderately correlated with grain and root Zn contents but exhibited a weak association with ZnHI, suggesting that Zn use efficiency is not solely governed by the redistribution of Zn within the plant. Correlation analysis confirmed that enhanced Zn availability from nano- and sulphate-based sources, improved grain Zn enrichment and PE efficiency. The observed Zn use efficiency improvement with nano-ZnO aligns with earlier reports on foliar biosynthesized Zn NPs [50], coated formulations [51], and integrated biofertilizer–nanofertilizer use [52]. Under drought and manure-amended conditions, ZnO NPs (~ 18 nm) significantly increased wheat biomass, chlorophyll, and Zn^2+^, Ca^2+^, and Mg^2+^ uptake, outperforming bulk ZnO at half the dose [53, 54]. Their ability to mitigate oxidative stress, enhance yield, and improve Zn accumulation confirms nano form of ZnO as efficient nanofertilizers offering higher uptake and targeted nutrient delivery for sustainable nutrient management [55, 56].

## Conclusion

This study addressed a critical knowledge gap concerning the comparative efficacy of different zinc (Zn) sources and doses applied as foliar sprays at key reproductive stages for enhancing Zn enrichment in rice. The experimental results demonstrated that Zn application, particularly through nano ZnO (ZnO NPs), significantly enhanced grain yield, Zn uptake, and Zn accumulation in rice grains. Above the yield and accumulation, the nano formulation exhibited promising effects on seed germination, seedling growth and vigour, chlorophyll content, and the regulation of defence-related enzyme activities in rice leaves. Additionally, ZnO NPs improved root length and enhanced Zn mobilisation and accumulation, thereby contributing to root-trait-mediated microbial activity and improved soil health. Under the experimental conditions of this study, foliar application of ZnO nanoparticles up to 250 mg L^− 1^ did not show visible phytotoxic effects in rice; however, further studies across different environments and growth stages are required to confirm the broader applicability of these findings. The absence of phytotoxic effects even at a concentration of 250 mg L^− 1^ further validates the safety and practical feasibility of this approach for Zn enrichment. Collectively, these findings suggest that ZnO NPs applied at optimal concentrations (150 to 250 mg L^− 1^) can serve as an effective alternative Zn carrier, offering agronomic, physiological, and nutritional benefits, and providing a sustainable alternative to conventional Zn fertilisers. However, future investigations on multi season and location field validation across diverse rice ecosystems, long-term impacts on soil microbial dynamics, environmental safety of nano-based Zn formulations and using advanced imaging and tracing techniques for Zn movement in a plant via soil/foliar route of application will be essential to support large-scale deployment and policy integration of nano-enabled Zn nutrition strategies in major crops.

## Data Availability

All data generated or analysed during this study are included in this published article.
